# New species of *Eriopeltastes* Burmeister & Schaum, 1840 (Coleoptera, Scarabaeidae, Cetoniinae, Trichiini) from South Africa

**DOI:** 10.3897/zookeys.422.7830

**Published:** 2014-07-03

**Authors:** Enrico Ricchiardi, Renzo Perissinotto

**Affiliations:** 1 Corso A. Tassoni 79/4, 10143 Torino, Italy; 2 Department of Zoology, Nelson Mandela Metropolitan University, P.O. Box 77000, Port Elizabeth 6031, South Africa

**Keywords:** Trichiini, new species, KwaZulu-Natal, mistbelt grassland, Karklook Nature Reserve, Blinkwater Nature Reserve

## Abstract

Both male and female of a new species of *Eriopeltasttes* Burmeister & Schaum, 1840, *E.* (*E.*) *ornatus* Ricchiardi, **sp. n.**, are described from the mistbelt grassland of KwaZulu-Natal. Sexual dimorphism is extreme in this genus, with females being brachypterous, fossorial and entirely black to dark brown in general body colour. This is only the fifth out of 12 known species in this genus for which the female is known. The species appears to be restricted to areas within or in the immediate vicinities of two marginal nature reserves, Karkloof and Blinkwater, in a grassland habitat that is regarded as one of the most threatened in the Province of KwaZulu-Natal. Despite its acknowledged importance as centre of endemism the area is currently in a precarious state of large-scale degradation.

## Introduction

As investigations progress in the more remote and previously neglected areas of the KwaZulu-Natal Province of South Africa, new taxa are being discovered on a regular basis. Often this occurs in places earmarked for conservation that are under threat of land use change, with potentially irreversible environmental impact. A satisfactory biodiversity census of this region is, unfortunately, still lacking, but is currently being pursued by the provincial conservation authority, Ezemvelo KwaZulu-Natal (EKZN) Wildlife. Within this programme, it is of fundamental importance that new species be described and their distribution mapped speedily in order to avoid compromising the viability of minor or secondary reserves where micro-endemic and threatened species may occur. The rapid reporting of previously unknown species will contribute to assessing the renewal of the protected status and potential consolidation of these reserves, under the auspices of the integrated Biodiversity Conservation Planning of EKZN Wildlife (Ricchiardi and Perissinotto 2013).

Since January 2000, a number of specimens of an undescribed species of *Eriopeltastes* Burmeister & Schaum, 1840 have been collected during investigations in and around two minor reserves with an uncertain future, Karklook and Blinkwater. Both are situated in the Midlands region of KwaZulu-Natal, which has undergone massive transformation during the past century, mainly as a result of afforestation for the timber and paper industry (Mucina and Rutherford 2006). Several invasive plant species have taken over large areas previously supporting indigenous vegetation, thus compounding the problem of habitat deterioration. The genus *Eriopeltastes* is endemic to South Africa and virtually all its known species are micro-endemic, mountain relicts extremely susceptible to habitat disturbance/destruction (Ricchiardi et al. 1999). A description of this new species is presented here, to provide among other things, support for the renewal of the protected status and consolidation of these secondary reserves.

## Methods

In the Karkloof Reserve, field collections were undertaken from January 2000 to January 2001 in the main section (R. Perissinotto & L. Clennell *legit*), and during November 2012 in the “Melmoth” section (A. Armstrong *legit*). At Blinkwater, a one time collection was made on 5 January 2012, on the main ridge just below its summit (A. Armstrong *legit*). On all occasions, specimens were either collected in flight using a standard net, or picked by hand while perched on grass blades or crawling on the ground. No special trapping devices were used.

The description of morphological characters follows the terminology used by Krikken (1984) and Ricchiardi et al. (1999). Specimen length was measured from the anterior margin of the clypeus to the apex of the pygidium. Specimen width represents the maximum width of the elytra. Photos of the holotype were taken with a Nikon Coolpix P7700 fixed to one of the eyepieces of a Wild dissecting microscope. Photos were processed with photo stacking technique, using Combine ZP (free software by Alan Hadley, http://www.hadleyweb.pwp.blueyonder.co.uk). Finally, the background was removed from the photos using Adobe Photoshop, in order to increase clarity of resolution.

The first author wrote the taxonomic part of this study, including the description of the new species. The second author contributed all natural history and ecological observations. Collections are abbreviated as follows: DMSA – Natural Science Museum, Durban, South Africa; ISAM – Iziko South African Museum, Cape Town, South Africa; SANC – South African National Collection of Insects, Pretoria, South Africa; TMSA – Ditsong National Museum of Natural History (formerly Transvaal Museum), Pretoria, South Africa; PCER – Private Collection Enrico Ricchiardi, Turin, Italy; PCRP – Private Collection R. Perissinotto & L. Clennell, Port Elizabeth, South Africa. Geographic abbreviations: ECA – Eastern Cape Province; FRS – Free State Province; KZN – Kwa-Zulu Natal Province; MPU – Mpumalanga Province.

## Taxonomic account

### Eriopeltastes (Eriopeltastes) ornatus

Taxon classificationAnimaliaColeopteraScarabaeidae

Ricchiardi sp. n.

http://zoobank.org/20E676E6-4586-4476-AFE1-7824F1BF2467

Figures 1 – 3

#### Type series.

Holotype (HT) ♂: South Africa, KZN, Karkloof Nature Reserve, 6.II.2000, R Perissinotto & L Clennell *legit* (ISAM). Paratypes: 6 ♀ same data as HT, but 22-23.I.2000 (ISAM, PCRP); 6 ♂ 2 ♀: same data as HT (TMSA, PCER, PCRP); 16 ♂ 1 ♀, same data as HT but 26-27.II.2000 (DMSA, PCER, PCRP); 1 ♂, same data as HT but 28.I.2001 (PCRP); 1 ♂, Karkloof NR Melmoth Section, 5.XI.2012, A Armstrong *legit* (SANC); 3 ♂, Blinkwater Ridge, 30.I.2012, A Armstrong *legit* (DMSA, PCRP, PCER).

#### Diagnosis.

**Figure 1.**
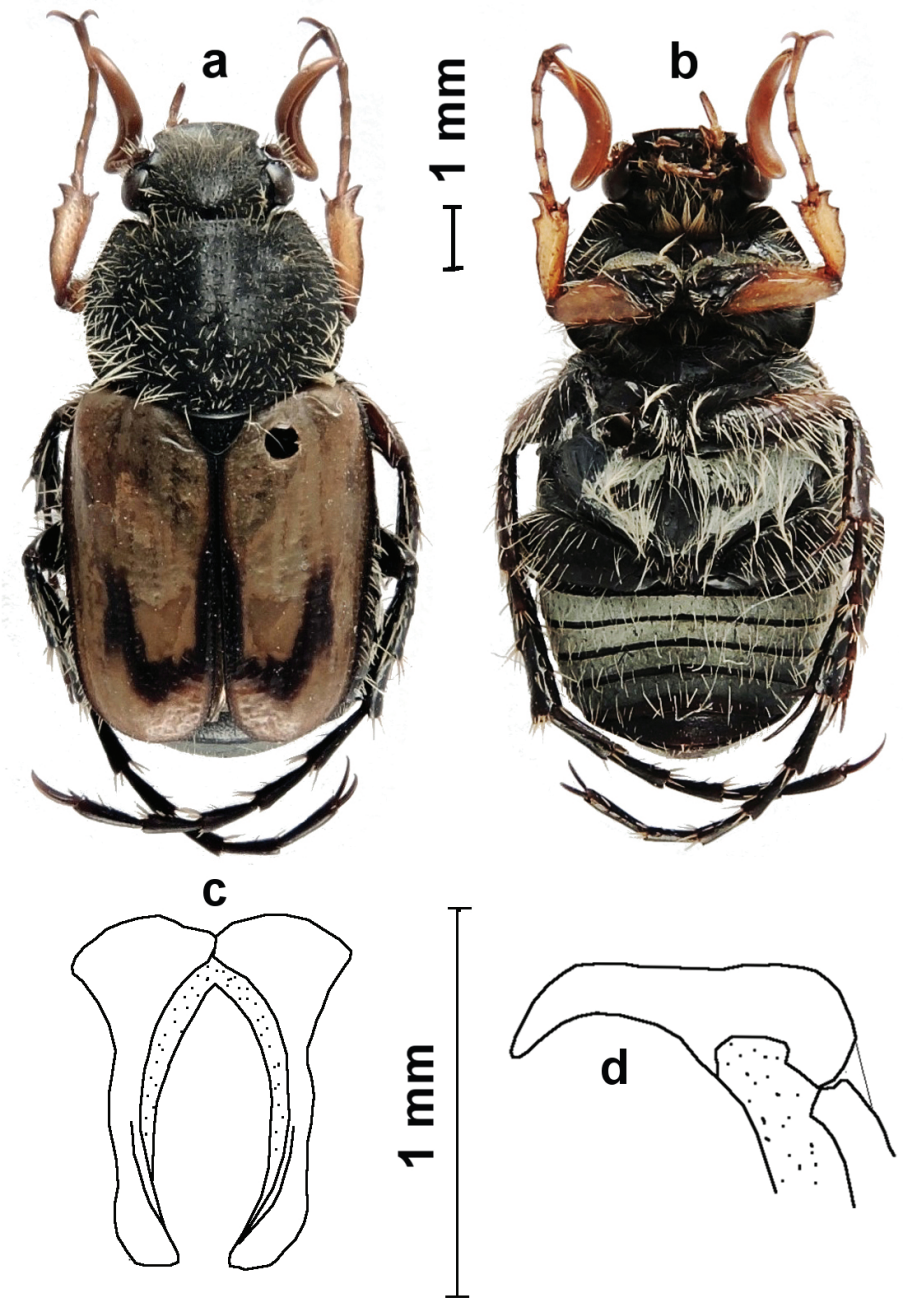
*Eriopeltastes (Eriopeltastes) ornatus* Ricchiardi, sp. n. Holotype ♂: **a** habitus in dorsal view **b** habitus in ventral view **c** parameres in frontal view **d** parameres in lateral view (Photo & drawing: Enrico Ricchiardi).

*Eriopeltastes (Eriopeltastes) ornatus* Ricchiardi, sp. n. is closest to *Eriopeltastes (Eriopeltastes) lineatus* Ricchiardi 1997. Both species have in common long and strongly curved (c-shaped) antennal clubs. They can, however, be easily separated as *Eriopeltastes (Eriopeltastes) ornatus* not only is much smaller in size than *Eriopeltastes (Eriopeltastes) lineatus*, but also exhibits the discal-lateral costa raised in the posterior half only. In *Eriopeltastes (Eriopeltastes) lineatus*, on the other hand, this costa is visibly raised throughout its length. The raised part of the discal-lateral costa is black in both species. The parameres are very similar in the two species, but differ significantly at the apex, which is slightly more expanded and curved downwards in *Eriopeltastes (Eriopeltastes) ornatus* Ricchiardi, sp. n. (Figure 1c; Ricchiardi 1997, Figures 3d & 3e).

#### Etymology.

The species is named after the prominent J-shaped black band it exhibits on the sutural margin and on the apical raised part of the costal disc of each elytron.

#### Description

**HT** ♂. Length 9.6 mm; width 4.6 mm. Body black; elytra light brown, with sutural margins black, black band continuing around posterior margin of elytral disk crossing apical umbone and extending anteriorly about ½ length of elytra (Figure 1a).

*Head*. Black, slightly shiny; wider than long; with dense punctuation on frons and vertex but poorly developed on clypeus; with scattered, testaceous, erected setae; eye canthus with long, scattered, testaceous setae; clypeus deeply concave, transverse, with front and side margins sharply raised, anterior margin rounded; antennae testaceous; club strongly C-shaped, more than twice as long as clypeus.

*Pronotum*. Black, shiny, glabrous; shape trapezoidal, with sides ridged but not crenulated; basal corners rounded, basal margin laterally sinuate, ridged basolaterally only; sides with white tomentum band approximating basal corner; midline grooved and covered internally with white tomentum, particularly at base; additional small tomentose area present on each side of disc; surface entirely covered with dense, long, testaceous, erect setae.

*Scutellum*. Black, glabrous, semicircular, enlarged; shiny, with scattered, round punctures.

*Elytra*. Testaceous, glabrous, shiny, with a round opaque area on the posterior margin of disc; apex rounded; with juxtasutural area black and posterior half of disco-lateral costa raised and black; behind disc a C-shaped black band joins juxtasutural and disco-lateral costae; striae poorly indicated; interstrie with large but shallow and scattered round punctures.

*Pygidium*. Semicircular, much wider than long, slightly constricted at sides; black, with erect, scattered black setae on disc; white tomentum covering entire surface, except area from middle of disc to apex.

*Ventral surface*. Black, covered with very scattered, long, flattened testaceous setae; abdominal sternites covered in white tomentum, except anal; tergites covered with white tomentum except at posterior edge; mesosternal protrusion absent (Figure 1b).

*Legs*. Protibiae testaceous, mesotibiae and metatibiae dark brown; with scattered, locally denser, long, erect, testaceous setae; protibiae with two external denticles; first protarsomere longer than second.

#### Description

**Figure 2.**
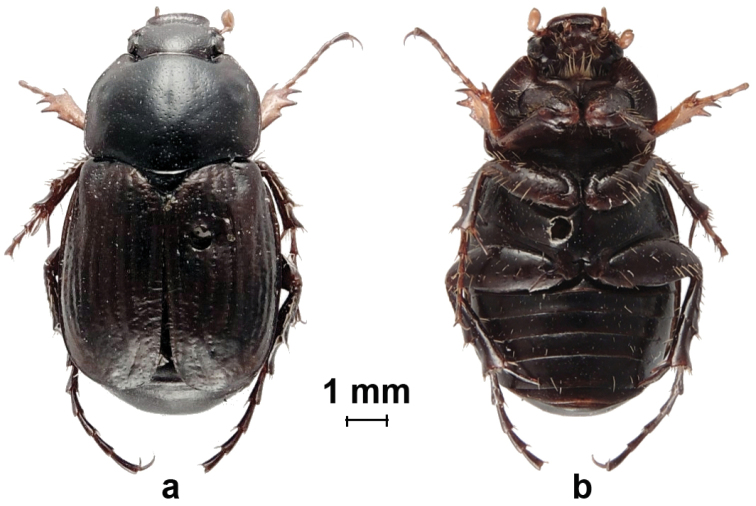
*Eriopeltastes (Eriopeltastes) ornatus* Ricchiardi, sp. n. Paratype ♀: **a** habitus in dorsal view **b** habitus in ventral view (Photo: Enrico Ricchiardi).

**PT** ♀. Length 10.1 mm; width 5.6 mm. Body black to dark brown, shiny, glabrous (Figure 2a).

*Head*. Black, poorly punctured, shiny, glabrous; clypeus concave, apex rounded, deflected and somewhat enlarged; antennae completely testaceous; club rounded, shorter than clypeal length.

*Pronotum*. Black, shiny, glabrous, trapezoidal, with sides parallel at centre and non-crenate; hind corners strongly rounded; hind margin centrally rounded, not ridged at middle and diverging smoothly towards lateral margins; with distinguishable rounded lateral impression at middle of lateral margins; covered with large but shallow, scattered punctures.

*Scutellum*. Black, glabrous, shiny, semicircular, wider than long, covered with deep, very scattered punctures.

*Elytra*. Apex rounded, dark brown, glabrous, shiny; striae marked with large, shallow, punctures; interstriae almost flat, with very scattered, small, shallow punctures.

*Pygidium*. Black, narrowing toward apex, laterally ridged, with large depression at middle of each side to apex; glabrous, shiny, black, with scattered shallow punctures.

*Ventral surface*. Black to dark brown; with very few, short and scattered testaceous setae; without any white tomentum (Figure 1b).

*Legs*. Generally as in male, with all tarsi slightly shorter; protibia broader than in male and exhibiting three denticles, with proximal smaller than other two.

#### Remarks.

The male paratypes are similar to the holotype in general appearance, but there is a range of variability in the extent of white tomentum markings on the pronotum and the width/length of the black band on the disco-lateral elytral costae. In extreme cases, the pronotum can be entirely black, without visible tomentose areas, while the elytra can appear completely pale testaceous, with black band virtually obsolete. All females are morphologically very similar, with ground colour from black to dark brown (Figure 2).

### *Eriopeltastes* updated species list

*Eriopeltastes (Parapeltastes) clarki* Ricchiardi, 2004: ECA

*Eriopeltastes (Eriopeltastes) clennelli* Ricchiardi, 1999: ECA

*Eriopeltastes (Eriopeltastes) evansi* Ricchiardi, 1997: MPU

*Eriopeltastes (Eriopeltastes) leucoprymnus* Burmeister & Schaum, 1840: FRS, KZN, MPU

*Eriopeltastes (Eriopeltastes) lineatus* Ricchiardi, 1997: KZN

*Eriopeltastes (Eriopeltastes) maculatus* Ricchiardi, 1999: KZN

*Eriopeltastes (Eriopeltastes) modestus* (Péringuey, 1907): MPU

*Eriopeltastes (Eriopeltastes) montanus* Ricchiardi, 1997: KZN

*Eriopeltastes (Eriopeltastes) natalensis* (Péringuey, 1907): KZN

*Eriopeltastes (Eriopeltastes) ntinini* Ricchiardi, 2013: KZN

*Eriopeltastes (Eriopeltastes) ornatus* Ricchiardi, sp. n.: KZN

*Eriopeltastes (Eriopeltastes) perissinottoi* Ricchiardi, 1999: ECA

### Updated key to the males of *Eriopeltastes*

**Table:** 

1	Clypeus oval, elongate; elytron red (subgenus *Parapeltastes*)	*Eriopeltastes clarki* Ricchiardi, 2004
–	Clypeus oval, widened; elytron testaceous (subgenus *Eriopeltastes*)	2
2	Antennal clubs straight	3
–	Antennal clubs curved, at least at apex	7
3	Antennal clubs not longer than 1.5 times the clypeal length	*Eriopeltastes perissinottoi* Ricchiardi, 1999
–	Antennal club 1.8–2.3 times longer than clypeal length	4
–	Antennal club at least 2.5 times longer than clypeal length	*Eriopeltastes natalensis* (Péringuey, 1907)
4	Front margin of clypeus straight	*Eriopeltastes evansi* Ricchiardi, 1997
–	Front margin of clypeus curved	5
5	Front margin of clypeus slightly notched at center	*Eriopeltastes ntinini* Ricchiardi, 2013
–	Front margin of clypeus regularly curved	6
6	Clypeal surface covered in small, scattered punctures	*Eriopeltastes montanus* Ricchiardi, 1997
–	Clypeal surface covered in large, thick punctures	*Eriopeltastes leucoprymnus* Burmeister & Schaum, 1840
7	Antennal clubs C-shaped	8
–	Antennal clubs curved at apex	9
8	Discolateral costa of elytron black and elevated on entire length	*Eriopeltastes lineatus* Ricchiardi, 1997
–	Discolateral costa of elytron elevated and black in posterior half only	*Eriopeltastes ornatus* Ricchiardi, sp. n.
9	Antennal clubs 1.8–2.3 times longer than clypeal length	*Eriopeltastes maculatus* Ricchiardi, 1999
–	Antennal clubs at least 2.5 times longer than clypeal length	*Eriopeltastes clennelli* Ricchiardi, 1999

## Discussion

There are currently 12 species described in the genus *Eriopeltastes* (Ricchiardi 1997, Ricchiardi et al. 1999, 2004; Ricchiardi and Perissinotto 2013) and 50% of these are endemic to the Province of KwaZulu-Natal. As females are brachypterous and unable to fly, species are generally very restricted in their distribution range, occupying elevations such as mountain slopes, hilltops and ridges (Ricchiardi et al. 1999). They are therefore of special biodiversity value, but unfortunately also very vulnerable to land use change and habitat degradation.

**Figure 3.**
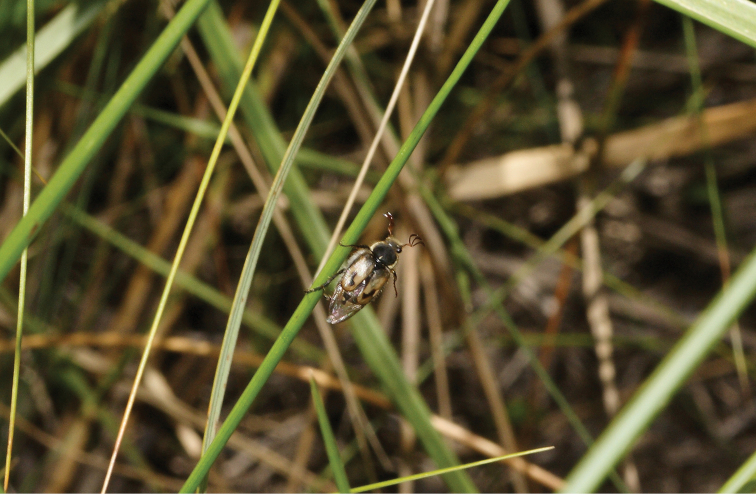
Male specimen of *Eriopeltastes (Eriopeltastes) ornatus* Ricchiardi, sp. n. in its grassland habitat on the slopes of Blinkwater Ridge (Photo: Adrian Armstrong).

*Eriopeltastes (Eriopeltastes) ornatus* Ricchiardi, sp. n., here described, is most closely related to *Eriopeltastes (Eriopeltastes) lineatus*, which is currently only known from few high altitude localities in the southern and central Drakensberg, namely Cobham, Giants Castle and Mdedelelo (Ricchiardi 1997, Ricchiardi et al. 1999). *Eriopeltastes (Eriopeltastes) ornatus* Ricchiardi, sp. n. occurs in the Midlands of KwaZulu-Natal, at altitudes ranging from approximately 1460 to 1664 m asl and in humid grassland habitats in the proximity of wetlands or streams. All specimens were collected during late morning to early afternoon hours (between 10:00 and 14:00) of sunny or partly cloudy days, immediately after major rainfall events. Typically, males fly fast and low just above the grass cover searching for pheromonal signals emitted by females on the ground (Figure 3). Females are generally occupied lying eggs, burrowing just below the surface of wet soil or crawling among grass tufts while searching for new suitable breeding areas. Occasionally, they have been observed also climbing up tall grass stems to bask in the late morning sun (R.P. pers. observ.). But it is not clear yet precisely what function this behaviour may play or facilitate. It seems likely that it may have something to do with their thermo-physiological balance, or with facilitating the emission of stronger and clearer pheromonal signals to guide searching males. It is also possible that this may even provide a take-off platform for short flights, although no female has been observed in flight yet.

**Figure 4.**
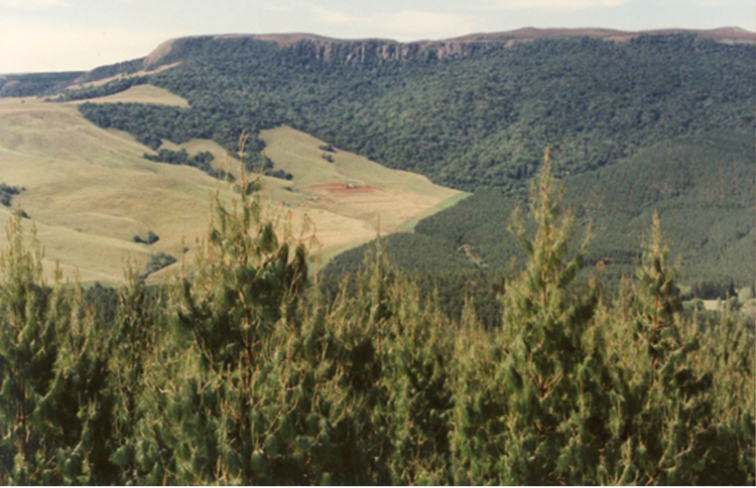
View of Karkloof, with grassland patches at the margin of mistbelt forest in the vicinities of the local colony of *Eriopeltastes (Eriopeltastes) ornatus* Ricchiardi, sp. n. (Photo: Kelson Camp).

**Figure 5.**
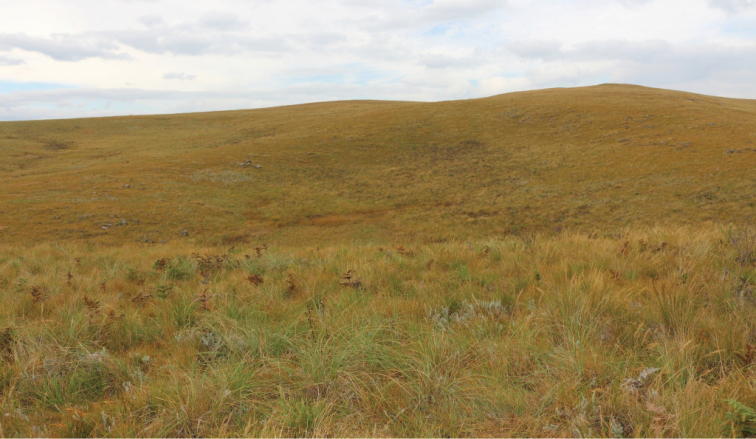
View of typical mistbelt grassland across the Blinkwater Ridge (Photo: Adrian Armstrong).

Unlike the Drakensberg range, which is currently adequately protected through the Maloti Drakensberg Transfrontier World Heritage Site and includes a Ramsar Wetland of International Importance, the KZN Midlands has been exposed to large-scale degradation, mainly through agroforestry, inappropriate cultivation, uncontrolled fires, overgrazing by livestock and invasion of alien vegetation (Low and Rebelo 1996). The natural vegetation types where *Eriopeltastes (Eriopeltastes) ornatus* Ricchiardi, sp. n. is found are classified as Mooi River Highland Grassland (type Gs 8) and Midlands Mistbelt Grassland (type Gs 9), which are part of the Sub-Escarpment Grassland Bioregion of the Grassland Biome (Mucina and Rutherford 2006). In the Karkloof Nature Reserve, rainfall averages over 1000 mm pa, with most occurring in summer. As a result, the western section of the reserve consists mainly of Southern Mistbelt Forest (type FOz 3), with patches of sourveld grassland interspersed with *Protea* tree species (Figure 4). The lower reaches of the Karkloof Nature Reserve and all of the Blinkwater Ridge exhibit predominantly Midlands Mistbelt Grassland, dominated by species such as *Themeda triandra* and *Aristida junciformis*, but also a large diversity of flowering plants (Figure 5) (Mucina and Rutherford 2006). This vegetation type is currently classified as “Endangered”, being regarded as one of the most threatened habitats in the Province of KwaZulu-Natal (Mucina and Rutherford 2006). The higher elevations of the Karkloof Nature Reserve are Mooi River Highland Grassland, which is classified as ‘Vulnerable’ because these grasslands are also under threat of extinction. According to the 2008 landcover map of KwaZulu-Natal (Ezemvelo KZN Wildlife 2011), only 24.5% of the original extent of the Midlands Mistbelt Grassland remained, much of it in a degraded condition, while 54.8% of the original extent of the Mooi River Highland Grassland remained, regardless of its condition.

Of the two localities where *Eriopeltastes (Eriopeltastes) ornatus* Ricchiardi, sp. n. is currently known to occur, one is part of provincial areas under statutory protection, the Karkloof Nature Reserve, while the other falls just outside the current borders of the Blinkwater Nature Reserve. The first is located approximately 19 km north of Howick, while the second lies 20 km southwest of Greytown. The two are less than 50 km apart, but the areas in between, as well as on each side, have undergone drastic land use changes. The Karkfloof Nature Reserve was formally proclaimed in July 1980 (Ordinance No. 76, Provincial Gazette of Natal No. 4185), while its eastern extensions and the Blinkwater Reserve were only proclaimed in August 2012 (Notice No. 83, Provincial Gazette of KwaZulu-Natal Vol. 6 No. 799). Challenges to the management of both nature reserves remain, due to a shortage of operational budgets.

The Mistbelt area of KwaZulu-Natal is increasingly being recognised as an important centre of endemism (van Wyk and Smith 2001), yet it is in a precarious state of large-scale degradation. The discovery of a Trichiini species new to science within or in the immediate vicinities of the Karkloof and Blinkwater reserves should contribute significantly to the biodiversity value of the region. It is thus important that the two nature reserves maintain their current statutory protection and possibly be earmarked for further expansion and consolidation. In particular, it would be important at this stage to secure the incorporation of the slope where the colony of *Eriopeltastes (Eriopeltastes) ornatus* occurs on the Blinkwater Ridge (Figure 5) into the adjacent Nature Reserve.

## Supplementary Material

XML Treatment for Eriopeltastes (Eriopeltastes) ornatus
